# Supplementary material for: Can co-ordination advancement of two-way FDI improve resource misallocation? Evidence from 285 cities in China
Source: PLoS One. 2024 Jul 8;19(7):e0304836. doi: 10.1371/journal.pone.0304836 (PMC11230532; doi:10.1371/journal.pone.0304836)
Supplement: Attachment — Submitted filename: Review comments.docx [file pone.0304836.s002.docx]

Dear author,：

we have the following opinions：

1.The analysis of labor force mismatch mechanism does not match the meaning of labor force mismatch

2. Lack of endogeneity testing

3. Heterogeneity can be achieved at different levels of coordinated promotion of bidirectional FDI
