# Supplementary material for: Can co-ordination advancement of two-way FDI improve resource misallocation? Evidence from 285 cities in China
Source: PLoS One. 2024 Jul 8;19(7):e0304836. doi: 10.1371/journal.pone.0304836 (PMC11230532; doi:10.1371/journal.pone.0304836)
Supplement: Attachment — Submitted filename: Response Reviewer.docx [file pone.0304836.s003.docx]

**Response to Reviewers**

First of all, we would like to thank reviewers for your insightful, constructive, and helpful comments on our manuscript entitled **“Can co-ordination advancement of two-way FDI Improve Resource Misallocation? Evidence from 285 Cities in China”** We have carefully considered and addressed all the comments and made necessary revisions in the revised manuscript. We provide a point-by-point response to the reviewers’ comments below.

The points raised by the reviewers are written in **bold font,** whereas our responses are shown in normal font, and the quotation of the revised manuscript is shown in red font.

# Reviewer #1:

1. **The analysis of labor force mismatch mechanism does not match the meaning of labor force mismatch.**

***Response:***

Thank you very much for your question. Firstly, we have carefully considered your question. Do you mean that there are issues with the labor force mismatch mechanism in our theoretical hypothesis section and the subsequent labor force mismatch. Based on our understanding, we have revised the research hypothesis section. In revised manuscript, we propose two new research hypotheses to make the logic clearer: The first hypothesis focuses on the mechanism of capital mismatch, and the second is the mechanism of labor mismatch. The results of using the labor distortion coefficient in robustness testing are inconsistent. We have rerun the code and made modifications to the text and table.

***…***

# 2 Theoretical analysis and research hypotheses

Misallocation refers to a deviation from the ideal state of resource configuration. Unlike efficient resource allocation, 'resource misallocation' denotes the presence of barriers to the free allocation of different resource elements between regions and industries, hindering the achievement of optimal allocation. Cheng [24] classified resource misallocation into "internal mismatch" and "external mismatch". Bai and Liu [8] argue that varying degrees of capital misallocation and labor resource misallocation exist in different regions of China, with significant regional disparities. The following theoretical perspective analyses the effect of co-ordination advancement of two-way FDI on resource misallocation.

In recent years, foreign direct investment (FDI) and outward foreign direct investment (OFDI) have experienced rapid growth as two major strategic pathways for China to acquire funds, technology, and market resources. The trend of co-ordination advancement of two-way FDI is increasingly prominent, serving as a significant driving force in guiding the rational flow of resources, improving resource misallocation, and enhancing resource allocation efficiency. Specifically, the co-ordination advancement of two-way FDI can improve capital, with the main pathway being the level of financial development. On one hand, during the early stages of development in developing countries, IFDI can alleviate the host country's capital needs and bring advanced technology and management expertise [25]. Through vertical and horizontal effects, it helps address financing constraints for enterprises in the host country, thereby reducing financing costs, enhancing capital liquidity, fostering a more open, inclusive, and efficient financial system, consequently improving capital misallocation, and enhancing capital allocation efficiency [26]. On the other hand, capital inflows also provide a solid foundation for outward investments [27]. The enhancement of the host country's financial strength and the improvement of its financial development level create necessary conditions for enterprises to engage in cross-border capital allocation and operations. Through outward foreign direct investment (OFDI) reverse technology spillover effects, financial institutions in the host country have established close connections with those in developed countries. Through cooperation, exchange, and competition with financial institutions in these developed countries, the management level and operational efficiency of financial institutions in the host country have been significantly enhanced. Therefore, the interaction between foreign direct investment (FDI) and outward foreign direct investment (OFDI) continuously promotes and guides the improvement of the host country's financial development level. The improvement of the financial development level can increase the liquidity of resources, reduce transaction costs of financial investment, thereby increasing investment, effectively alleviating capital misallocation, and enhancing capital allocation efficiency [28].

**H1**: Coordinated development in two-way FDI can enhance capital, primarily through advancements in the level of financial development.

Coordinated development in two-way FDI exacerbates the mismatch of labor resources by increasing labor costs. On one hand, IFDI increases the demand for labor in the host country, leading to changes in the geographical distribution of labor and exacerbating the issue of labor mismatch. At the same time, foreign enterprises will attract sustained labor inflows by raising labor prices (Sun and Wang, 2022) [29]. This has led to a phenomenon of 'labor shortages' among local enterprises, exacerbating labor scarcity. With the continuous influx of IFDI, the host country may face challenges of labor supply shortages, further exacerbating resource misallocation. On the other hand, with the continuous outflow of OFDI, it may lead to the emigration of labor from some developing countries. This emigration of labor mainly manifests in two aspects. Firstly, some enterprises will export a portion of their labor force through outward foreign direct investment (Lai et al., 2020) [30]. Secondly, outward foreign direct investment companies will dispatch management and technical personnel to overseas subsidiaries, inevitably creating some low-skilled job positions. This, in turn, further exacerbates the host country's demand for labor. Due to the inability of labor supply to meet rapidly growing demand in the short term, the rise in labor costs is actually detrimental to addressing the issue of labor mismatch. Therefore, two-way FDI exacerbates the degree of labor mismatch.

H2: Coordinated development of two-way FDI exacerbates labor mismatch, primarily through increasing labor costs.

***…***

## 4.3 Robustness tests

The capital deformation coefficient (*kcon*) and labor deformation coefficient (*lcon*) are calculated using Bai and Liu [8] methodology and capital deformation coefficients and labor deformation coefficients are used instead of capital resource misallocation and labor resource misallocation. The capital and labor deformation coefficients are calculated as:

$kcon=\frac{{MP}_{K}}{r}-1={\beta K}_{i}=\frac{p_{i}y_{i}}{{rk}_{i}}-1$	(9)

$lcon=\frac{{MP}_{L}}{w}-1={\beta L}_{i}=\frac{p_{i}y_{i}}{{w_{i}k}_{i}}-1$	(10)

*p_i_y_i_* is the total value of production; *r* is capital’s price. Hsieh and Klenow [5] a 10% value was obtained; *W_i_* is the price of labor in city *i.* MP_k_ and *MP_l_* are the marginal output of capital and labor, respectively.

Constructing the coefficient of capital distortion as well as the coefficient of labor distortion according to equations 9 and 10. Table 3 shows the calculation results of the impact of co-ordination advancement of two-way FDI on the deformation coefficient of capital and labor. Column (2) show the results of estimating the coefficient of capital distortion in the current and lagged a period for the co-ordination development of two-way FDI. It can be seen the negative coefficient impact on capital distortion is significant for both the current and lagged periods of co-ordination advancement of two-way FDI levels. This indicates that it can significantly alleviate the degree of capital misallocation. The last two columns show the impact of co-ordination advancement of two-way FDI on labor deformation coefficient during the current and lagging period. The results suggest that The research results indicate that the coordinated development level of current and lagged two-way FDI has a promoting effect on the coefficient of labor distortion.

**Table 3.** **Robustness test**

|  | *kcon* | *kcon* | *lcon* | *lcon* |
| --- | --- | --- | --- | --- |
|  | (1) | (2) | (3) | (4) |
| *dioc* | -0.0569^***^ |  | 0.0336^**^ |  |
|  | (0.0162) |  | (0.0149) |  |
| *L.dioc* |  | -0.0440^**^ |  | 0.0359^*^ |
|  |  | (0.0182) |  | (0.0184) |
| ln*pgdp* | 0.0171 | 0.0159 | 0.0524^***^ | 0.0519^***^ |
|  | (0.0108) | (0.0110) | (0.0070) | (0.0076) |
| *pub* | 0.0022 | 0.0028 | -0.0012 | -0.0036 |
|  | (0.0136) | (0.0140) | (0.0129) | (0.0135) |
| *fint* | 0.0099 | 0.0069 | -0.0374^***^ | -0.0343^***^ |
|  | (0.0095) | (0.0099) | (0.0114) | (0.0116) |
| ln*fin* | -0.0023 | 0.0003 | -0.0221^***^ | -0.0212^***^ |
|  | (0.0067) | (0.0069) | (0.0062) | (0.0062) |
| *_cons* | 1.1296^***^ | 1.0919^***^ | -0.0408^***^ | 1.0245^***^ |
|  | (0.1947) | (0.1988) | (0.0093) | (0.1471) |
| Year FE | Y | Y | Y | Y |
| City FE | Y | Y | Y | Y |
| *N* | 3810 | 3810 | 3570 | 3315 |
| adj. *R*^2^ | 0.699 | 0.716 | 0.746 | 0.750 |
| F | 3.0930 | 1.5379 | 244.3748 | 236.3851 |

Notes: Values in brackets indicate standard errors for city clustering; ^*^, ^**^, ^***^ indicate significance at 10%, 5%, and 1%, respectively, as shown below.

***…***

2. **Lack of endogeneity testing.**

***Response:***

Thank you very much for your question. The original manuscript lacks essential endogeneity testing analysis, which will be supplemented in the revised version. The revised manuscript employs two methods to examine endogeneity. The first method involves employing instrumental variable techniques, where instrumental variables are generated to exhibit a strong correlation with the coordinated development of co-ordination advancement of two-way FDI but a weak correlation with levels of resource misallocation (capital misallocation index and labor misallocation index). This approach effectively addresses endogeneity concerns. The second approach involves employing the System GMM method to test for endogeneity issues, utilizing the differences of lagged variables to control for serial correlation. By employing these two methods, endogeneity interference is addressed. The revised part is in “4.4 Endogeneity test”.

…

## 4.4 Endogeneity test

The co-ordination advancement of two-way FDI and resource misallocation are both important economic variables within the economic system, and their endogeneity is inevitable. That is co-ordination advancement of two-way FDI impacts resource misallocation, and vice versa. Furthermore, there are also issues of missing variables and measurement errors in the model, which are the main sources of endogeneity. To overcome the interference of potential endogeneity on the estimation results, this paper uses two approaches to deal with it:

First, the instrumental variables approach. Bartik [37] developed these instrumental variables. Specifically, the method involves multiplying the initial value of each city's degree of co-ordination advancement of two-way FDI by the growth rate of the national average degree of co-ordination advancement of two-way FDI relative to the initial period, resulting in the Bartik instrumental variable (*bartikiv*). This instrumental variable demonstrates a strong correlation with the co-ordination development of two-way FDI, but a relatively weak correlation with the level of resource misallocation (as measured by capital misallocation indices and labor misallocation indices). Table 4 presents the estimation results of the instrumental variables. Notably, there are no estimates indicating weak instrumental variables or overidentification in the first stage. Additionally, both the estimates of *bartikIV* and L.*bartikIV* are highly significant and positive. The findings from the second-stage estimation suggest that the co-ordination advancement of two-way FDI has a moderating effect on capital misallocation and exacerbates labor misallocation, consistent with the results obtained from the benchmark regression estimation.

**Table 4. Estimation of instrumental variables**

|  | *abstauk* | *abstauk* | *abstaul* | *abstaul* |
| --- | --- | --- | --- | --- |
|  | (1) | (2) | (3) | (4) |
| *dioc* | -0.3496^***^ |  | 0.1585^***^ |  |
|  | (0.0344) |  | (0.0387) |  |
| L.*dioc* |  | -0.3528^***^ |  | 0.1456^***^ |
|  |  | (0.0340) |  | (0.0393) |
| ln*pgdp* | 0.0495^***^ | 0.0354^***^ | 0.0258^**^ | 0.0330^***^ |
|  | (0.0091) | (0.0085) | (0.0102) | (0.0098) |
| *pub* | -0.0130 | -0.0179^*^ | -0.0356^***^ | -0.0313^***^ |
|  | (0.0092) | (0.0092) | (0.0104) | (0.0107) |
| *fint* | 0.0161^**^ | 0.0142^**^ | -0.0198^***^ | -0.0139^*^ |
|  | (0.0064) | (0.0064) | (0.0072) | (0.0074) |
| ln*fin* | 0.0319^***^ | 0.0262^***^ | 0.0006 | 0.0052 |
|  | (0.0044) | (0.0047) | (0.0050) | (0.0054) |
| Year FE | Y | Y | Y | Y |
| City FE | Y | Y | Y | Y |
| *N* | 3810 | 3556 | 3810 | 3556 |
| The first stage |  |  |  |  |
| *bartikiv* | 0.6665^***^ |  | 0.6665^***^ |  |
|  | (0.0429) |  | (0. 0429) |  |
| L.*bartikiv* |  | 0.7142^***^ |  | 0.7142^***^ |
|  |  | (0.0446) |  | (0.0446) |
| Underidentification test (Anderson canon. corr. LM statistic) | 226.569 | 239.181 | 226.569 | 239.181 |
| Weak identification test (Cragg-Donald Wald F statistic) | 240.694 | 256.454 | 240.694 | 256.454 |

Notes: The table displays regression coefficients with standard errors in parentheses. Statistical significance is denoted by *, **, ***, which correspond to p < 0.1, p < 0.05, and p < 0.01, respectively. This notation is consistent throughout the text.

Second, system GMM estimation is utilized. Systematic GMM estimation can incorporate lagged explanatory variable and instrumental variables effectively, enabling the selection of endogenous variables, making it a useful method for addressing potential endogeneity. Employing the system GMM approach, this paper utilizes the *xtdpdsys* command, considering *dioc* as an endogenous variable. The results of the estimation using the two-step method are presented in Table 5. The findings indicate that the coefficients of the previous timeframe for both capital misallocation and labor resource misallocation are significantly positive, suggesting a path dependence between capital misallocation and labor resource misallocation over time. The calculated coefficients reveal a notably negative correlation between capital misallocation and both the coordination advancement of two-way FDI and the lagged one-period coordination development of two-way FDI. Additionally, the estimated coefficients on labor misallocation are significantly positive for both the coordination advancement of two-way FDI and the coordination advancement of two-way FDI with a one-period lag. This is consistent with the initial estimates, and H1 is confirmed.

**Table 5. System GMM estimation**

|  | *abstauk* | *abstauk* | *abstaul* | *abstaul* |
| --- | --- | --- | --- | --- |
|  | (1) | (2) | (3) | (4) |
| L.*abstauk* | 1.1121^***^ | 1.0951^***^ |  |  |
|  | (0.0040) | (0.0057) |  |  |
| L.*abstaul* |  |  | 0.9636^***^ | 1.0157^***^ |
|  |  |  | (0.0013) | (0.0045) |
| *dioc* | -0.0168^***^ |  | 0.0358^***^ |  |
|  | (0.0022) |  | (0.0009) |  |
| L.*dioc* |  | -0.0081^**^ |  | 0.0105^**^ |
|  |  | (0.0034) |  | (0.0053) |
| *trade* | 0.0114^***^ | 0.0135^***^ | -0.0012 | 0.0057^*^ |
|  | (0.0018) | (0.0026) | (0.0011) | (0.0029) |
| *book* | 0.0087^***^ | 0.0105^***^ | -0.0089^***^ | 0.0221^***^ |
|  | (0.0024) | (0.0027) | (0.0014) | (0.0053) |
| *fint* | -0.0035^***^ | 0.0030^**^ | 0.0043^***^ | 0.0001 |
|  | (0.0009) | (0.0015) | (0.0013) | (0.0031) |
| *scie* | 0.0010 | -0.0001 | 0.0046^***^ | 0.0038 |
|  | (0.0011) | (0.0008) | (0.0005) | (0.0023) |
| _cons | -0.1690^***^ | -0.3007^***^ | -0.0408^***^ | -0.1880^***^ |
|  | (0.0259) | (0.0372) | (0.0093) | (0.0408) |
| AR (1) P | 0.000 | 0.000 | 0.000 | 0.0000 |
| AR (2) P | 0.2041 | 0.2446 | 0.2080 | 0.2226 |
| Sargan P | 0.1887 | 0.1491 | 0.1837 | 0.1791 |
| *N* | 3556 | 3556 | 3556 | 3556 |

…

3. **Heterogeneity can be achieved at different levels of coordinated promotion of bidirectional FDI.**

***Response:***

Thank you very much for your question. In the original manuscript, the examination of heterogeneity was conducted based on urban geographic locations and urban development levels, overlooking the impact of varying degrees of co-ordination advancement of two-way FDI on resource allocation.

In response to your feedback, we grouped cities based on different levels of co-ordination advancement of two-way FDI. Cities were divided into two groups: the first comprising cities with co-ordination advancement of two-way FDI levels above the average, and the second comprising cities with levels below the average. Regression analyses were then conducted within each group, and the obtained results remained significant. The revised part is in “4.5 Heterogeneity test”.

***…***

First, the heterogeneity of the level of co-ordination advancement in two-way FDI, Chinese cities are classified based on the level of coordination advancement in two-way FDI, and the disparities in this regard are estimated. The first and third columns indicate that when the level of coordination advancement in two-way FDI exceeds the average, it significantly improves capital mismatch while significantly exacerbating labor mismatch. This finding aligns with the benchmark regression results. The second and fourth columns show that when the level of co-ordination advancement in two-way FDI is lower than the average level, the impact on resource allocation is insignificant due to the low level of co-ordination advancement in two-way FDI.

**Table 5. Two-way FDI coordinated development level Heterogeneity**

|  | *abstauk* | | *abstaul* | |
| --- | --- | --- | --- | --- |
|  | High-coordination | Low-coordination | High coordination | Low-coordination |
|  | (1) | (2) | (3) | (4) |
| *lndioc* | -0.0817^***^ | 0.0023 | 0.1759^***^ | 0.0212 |
|  | (0.0308) | (0.0078) | (0.0321) | (0.0187) |
| *trade* | 0.0029 | 0.0029 | 0.0161 | 0.0344^***^ |
|  | (0.0172) | (0.0072) | (0.0203) | (0.0126) |
| *book* | -0.0316^***^ | -0.0150^**^ | -0.0172 | -0.0133 |
|  | (0.0114) | (0.0076) | (0.0173) | (0.0140) |
| *fint* | -0.0014 | -0.0055 | -0.0310^**^ | 0.0142 |
|  | (0.0086) | (0.0046) | (0.0140) | (0.0092) |
| *scie* | 0.0121 | -0.0057 | -0.0126^**^ | 0.0395 |
|  | (0.0086) | (0.0055) | (0.0060) | (0.0252) |
| *_cons* | 0.9446^***^ | 0.3208^***^ | -0.4395 | -0.1410 |
|  | (0.2947) | (0.1185) | (0.4188) | (0.1973) |
| Year FE | Y | Y | Y | Y |
| City FE | Y | Y | Y | Y |
| N | 1882 | 1889 | 1882 | 1889 |
| adj. *R*^2^ | 0.8245 | 0.6307 | 0.7282 | 0.7260 |
| F | 3.6290 | 1.2486 | 6.8381 | 4.2513 |

***…***

# Reviewer #2:

1. **The abstract needs to be rewritten to systematically provide a proper summary of the study touching on the objective, the method, the timeframe, the findings, the conclusion and recommendations.**

***Response:***

Thank you very much for your valuable suggestion. Due to the lack of essential information in the abstract as required. In the revised manuscript, we have reorganized the language and added detailed information. These details encompass the research objectives, methods, timeframe, findings, conclusions, recommendations, and have also involved updating some of the literature references. The revised part is in “1 Introduction”.

***…***

# 1 Introduction

Since the turn of the century, under the guidance of "bringing in" and "going out", the scope of China's outward foreign direct investment (OFDI) and international foreign direct investment in China (IFDI) has rapidly expanded. Based on the World Investment Report 2021, China utilized IFDI flows, reaching $144.4 billion, and OFDI flows, touching $153.7 billion, with IFDI and OFDI stocks accounting for 4.6% and 5.99% correspondingly by the end of 2020. Coordinated development of two-way foreign direct investment (FDI) is an important way of resource flow, connecting two markets and two types of resources, playing a crucial role in extending the industrial chain and promoting industrial upgrading. The co-ordination advancement of two-way FDI is conducive to the long-term sustainable development of developing countries, and such inclusive growth will strongly promote the optimal allocation of resources and rapid economic growth [1] China has guided IFDI into the market and led to a surge in labor demand and increased resource supply. At the same time, by integrating into the global industrial chain through OFDI, it guides domestic resources to flow abroad and affects the demand for domestic labor market. Therefore, it is inadequate to study the impact of IFDI or OFDI on resource misallocation solely from its perspective. It is crucial to combine both and study the co-ordination development of two-way FDI.

Due to long-term imbalanced developmental model, the issue of resource misallocation in China has always been a focus of attention in the academic community. With the swift expansion of the economy, the coarse economic growth pattern has resulted in the low efficiency in resource allocation [2]. The nature of the diffusion and transmission of resource allocation distortions has resulted in structural problems in Chinese economy [3], which deviates from the effective allocation of resources and obstructs the China’s economic advancement. The report of the 20th Party Congress states ‘Combining the implementation of the domestic demand expansion strategy with deepening of supply side structural reforms’, which is an unavoidable decision to hasten the formation of a novel development model with a focus on the domestic macro-cycle and mutually reinforcing domestic and global double-cycles. In the context of double-cycling, co-ordination advancement of two-way FDI promotes the optimal allocation of domestic resources [4]. Foreign and domestic multinational corporations invest their resources to where they yield the highest returns, forming a co-ordination development of two-way FDI. Co-ordination advancement of two-way FDI has ability to integrate domestic and international resources, which can provide impetus for supply side structural reforms. In a narrow sense, resources are divided into capital and labor. The study about the misallocation of capital and labor resources of co-ordination advancement of two-way FDI is a question worthy of in-depth research.

Currently, scholars have dedicated significant attention to co-ordination advancement of two-way FDI and the problem of resource misallocation. Literature relevant to this paper encompasses two main areas: Firstly, the measurement of resource misallocation and the factors influencing them. Academics mainly measured resource misallocation in both micro and macro dimensions. At the micro level, there are mainly parametric and non-parametric methods. The parametric approach measure is to measure the extent of resource misallocation through the actual and effective output gap [5]. The non-parametric method combines resource flow with enterprise size and productivity level, and decomposes factor productivity through OP technology to obtain covariates that reflect the degree of resource misallocation [6]. At the macro level, Chen and Hu [7] used the absolute and relative deformation coefficients of capital prices to measure capital misallocation, providing scholars with ideas for later measuring resource misallocation; Bai and Liu [8] used urban data to construct the C-D production function and used regression method to calculate the capital misallocation and labor resource misallocation exponent at the China’s urban level. Scholars have primarily measured capital misallocation and labor resource misallocation. The main drivers of capital misallocation are monopoly frictions and financial frictions [9], the development of information technology [10], administrative friction [11], financing constraints [12], information asymmetry [13], stakeholder monitoring of firms [14]; The main driving factors driving labor misallocation are monopoly frictions [15], High-speed railway opening [16], labor skill levels [17], decision-making in the marketplace [18], increased Regulation of Businesses [19]]. Secondly, the measurement of the degree of co-ordination advancement of two-way FDI and its implications. There are two existing measures in the literature to calculate the level of co-ordination development of two-way FDI. Firstly, is to adopt a two-way FDI interaction term, secondly is to calculate the degree of coordination of two-way FDI. The impact of co-ordination development of two-way FDI, focusing mainly on green development [20], green technology innovation efficiency [21], carbon emission reduction [22], quality economic growth [23]. From the above, extensive literature has been developed on resource misallocation and co-ordination development of two-way FDI. However, there is no literature that incorporates co-ordination advancement of two-way FDI and resource misallocation into the same theoretical framework.

In light of this, the paper empirically examines the impact of coordinated development of two-way FDI on resource misallocation using panel data from 285 prefectural-level cities in China spanning from 2006 to 2020. Striving to contribute to enriching related research and offering reasonable policy recommendations. Moreover, the endogeneity issue was addressed by employing instrumental variable method and system GMM model. Furthermore, the study divided the stages of coordinated development of two-way FDI based on its level, exploring the impact of different stages of coordinated development of two-way FDI on resource allocation; And the impact of coordinated development of two-way FDI on resource misallocation was examined based on the development levels and geographical locations of various cities. Finally, the transmission mechanism of the impact of coordinated development of two-way FDI on resource misallocation was discussed through the pathways of deepening financial development and rising labor costs

The results show that the coordinated development of two-way FDI optimizes capital allocation by enhancing the level of financial development. However, by increasing labor costs, it exacerbates labor misallocation. Despite a series of robustness checks, the results remain stable. Based on empirical evidence, the conclusion is drawn: China is currently in a high-quality development stage transitioning from factor input to improving resource allocation efficiency. It should unwaveringly adhere to its strategy of opening-up to the outside world, encourage enterprises to actively 'bring in' and 'go out,' and strengthen macro-control. Relying on the coordinated development of two-way FDI to achieve optimized resource allocation and high-quality economic development.

Distinguished from previous research, the main contribution of this study may lie in: Firstly, integrating the coordinated development of two-way FDI with resource misallocation into the same theoretical framework enriches the relevant research; Secondly, overcoming endogeneity issues through the use of instrumental variable methods and the system GMM model; Thirdly, conducting heterogeneous analysis from different perspectives to ensure the robustness of the results, which contributes to more precise formulation of policy pathways for improving resource misallocation; Fourthly, the utilization of a mediation effects model to examine the impact of coordinated development of two-way FDI on resource misallocation, leading to a deeper understanding of the underlying causes of resource misallocation.

The paper is structured as follows: in the second part embodies discussion and hypotheses, followed by analytical methods and data collection in the third part, the empirical testing steps, and analysis in the fourth part, the fifth part is mechanism analysis, and the finally, conclusions and recommendations in the last part.

***…***

2. **The manuscript requires language editing.**

***Response:***

Thank you very much for your valuable suggestion. Based on your suggestions, we have corrected misleading expressions as far as possible and fixed all grammatical errors in the original text.

3. **Abstauk and abstaul are not explanatory variables in equations 1 and 2.**

***Response:***

Thank you very much for your valuable suggestion. We apologize for our carelessness. The original manuscript misinterprets the explanations for *Abstauk* and *Abstaul* in equations 1 and 2. According to the equations, *Abstauk* and *Abstaul* should be the dependent variables in equations 1 and 2. Representing the capital misallocation index and the labor misallocation index in urban areas. The revised part is in “3.1 Model Settings”.

…

## 3.1 Model Settings

Drawing on Bai and Liu (2018) [8], the formula is developed in the study to co-ordination advancement of two-way FDI’s influence on capital misallocation and labor resource misallocation:

${abstauk}_{it}=\alpha+\beta{dioc}_{it}+\tau X_{it}+\delta_{i}+\sigma_{t}+\varepsilon_{it}$	(1)

${abstaul}_{it}=\alpha+\beta{dioc}_{it}+\tau X_{it}+\delta_{i}+\sigma_{t}+\varepsilon_{it}$	(2)

Here，i denotes city and t denotes year; abstauk_it_, abstaul_it_ are the explained variables, denoting the capital misallocation and labor misallocation indices for city i in year t, respectively. The core explanatory variable is expressed as the coupled coordination exponent of two-way FDI. X_it_ denotes control variables, $\delta_{i}$ denotes individual fixed effects, $\sigma_{t}$ denotes year fixed effects, and $\varepsilon_{it}$ is a residual term. $\beta$ as the coefficient of interest in this paper, if it is significantly negative, it indicates that co-ordination advancement of two-way FDI has a positive effect on resource misallocation but vice versa worsens the level of resource misallocation.

…

4. **The econometric model (including all the control variables) that was eventually estimated is not provided.**

***Response:***

Thank you very much for your valuable suggestion. In the original manuscript, the section on baseline regression lacks the results after controlling for variables, which will be added in the revised version. The revised part is in “4.1 Benchmark regression”.

***…***

This paper's data are panel data. Drawing on [36], Before conducting empirical research, we have to use the Hausman test to choose the choice between fixed and random effects. The result shows is chi2 (5) = 46.41, Prob > chi2 = 0.000, we need to use a fixed effect.

…

5. **IFDI is not clearly defined, does this refer to FDI inflows into China?**

***Response:***

Thank you very much for your valuable question. In the original manuscript, there was a lack of precise definition for IFDI. In the introduction section, we have supplemented the precise definitions for both IFDI and OFDI. The revised part is in “3.2 Variable Declaration”.

…

Outward foreign direct investment (OFDI) refers to the transfer of capital from the home country to the host country for production and sales by the mode of overseas operation and management. In this paper, it refers to China's foreign direct investment; Inward foreign direct investment (IFDI) refers to the process by which foreign enterprises or individuals inject capital, technology, management experience and other resources into the economic system of a specific country. In this paper, it refers to the investment made by foreign enterprises or individuals in China.

…

6. **The study does not properly explain the theoretical link between fdi flows and resource misallocation. This should be the corner stone of this study.**

***Response:***

Thank you very much for your valuable suggestion. In the original manuscript, there was insufficient explanation regarding the connection between co-ordination advancement of two-way FDI and resource misallocation, resulting in a lack of theoretical foundation throughout the article. Here, we provide a thorough explanation of the theoretical analysis and research hypotheses raised in response to your question. In the revised manuscript, the research hypotheses and theoretical analysis are divided into two categories: The first category posits that the coordinated development of co-ordination advancement of two-way FDI improves capital resource misallocation by enhancing financial development. The second category suggests that the coordinated development of co-ordination advancement of two-way FDI exacerbates labor resource misallocation by increasing labor costs. The revised part is in “2 Theoretical analysis and research hypotheses”.

***…***

# 2 Theoretical analysis and research hypotheses

Misallocation refers to a deviation from the ideal state of resource configuration. Unlike efficient resource allocation, 'resource misallocation' denotes the presence of barriers to the free allocation of different resource elements between regions and industries, hindering the achievement of optimal allocation. Cheng [24] classified resource misallocation into "internal mismatch" and "external mismatch". Bai and Liu [8] argue that varying degrees of capital misallocation and labor resource misallocation exist in different regions of China, with significant regional disparities. The following theoretical perspective analyses the effect of co-ordination advancement of two-way FDI on resource misallocation.

In recent years, foreign direct investment (FDI) and outward foreign direct investment (OFDI) have experienced rapid growth as two major strategic pathways for China to acquire funds, technology, and market resources. The trend of co-ordination advancement of two-way FDI is increasingly prominent, serving as a significant driving force in guiding the rational flow of resources, improving resource misallocation, and enhancing resource allocation efficiency. Specifically, the co-ordination advancement of two-way FDI can improve capital, with the main pathway being the level of financial development. On one hand, during the early stages of development in developing countries, IFDI can alleviate the host country's capital needs and bring advanced technology and management expertise [25]. Through vertical and horizontal effects, it helps address financing constraints for enterprises in the host country, thereby reducing financing costs, enhancing capital liquidity, fostering a more open, inclusive, and efficient financial system, consequently improving capital misallocation, and enhancing capital allocation efficiency [26]. On the other hand, capital inflows also provide a solid foundation for outward investments [27]. The enhancement of the host country's financial strength and the improvement of its financial development level create necessary conditions for enterprises to engage in cross-border capital allocation and operations. Through outward foreign direct investment (OFDI) reverse technology spillover effects, financial institutions in the host country have established close connections with those in developed countries. Through cooperation, exchange, and competition with financial institutions in these developed countries, the management level and operational efficiency of financial institutions in the host country have been significantly enhanced. Therefore, the interaction between foreign direct investment (FDI) and outward foreign direct investment (OFDI) continuously promotes and guides the improvement of the host country's financial development level. The improvement of the financial development level can increase the liquidity of resources, reduce transaction costs of financial investment, thereby increasing investment, effectively alleviating capital misallocation, and enhancing capital allocation efficiency [28].

**H1**: Coordinated development in two-way FDI can enhance capital, primarily through advancements in the level of financial development.

Coordinated development in two-way FDI exacerbates the mismatch of labor resources by increasing labor costs. On one hand, IFDI increases the demand for labor in the host country, leading to changes in the geographical distribution of labor and exacerbating the issue of labor mismatch. At the same time, foreign enterprises will attract sustained labor inflows by raising labor prices (Sun and Wang, 2022) [29]. This has led to a phenomenon of 'labor shortages' among local enterprises, exacerbating labor scarcity. With the continuous influx of IFDI, the host country may face challenges of labor supply shortages, further exacerbating resource misallocation. On the other hand, with the continuous outflow of OFDI, it may lead to the emigration of labor from some developing countries. This emigration of labor mainly manifests in two aspects. Firstly, some enterprises will export a portion of their labor force through outward foreign direct investment (Lai et al., 2020) [30]. Secondly, outward foreign direct investment companies will dispatch management and technical personnel to overseas subsidiaries, inevitably creating some low-skilled job positions. This, in turn, further exacerbates the host country's demand for labor. Due to the inability of labor supply to meet rapidly growing demand in the short term, the rise in labor costs is actually detrimental to addressing the issue of labor mismatch. Therefore, two-way FDI exacerbates the degree of labor mismatch.

H2: Coordinated development of two-way FDI exacerbates labor mismatch, primarily through increasing labor costs.

***…***

7. **Which cities were covered in the study?**

***Response:***

Thank you very much for your valuable question. To maintain the fluidity, readability, and aesthetic appeal of the text, the cities covered by the study were not individually listed. Considering the availability of data, the final dataset of this study only includes 285 prefecture-level cities in China.

Here, the cities included in the study are listed: Beijing, Tianjin, Shijiazhuang, Tangshan, Qinhuangdao, Handan, Xingtai, Baoding, Zhangjiakou, Chengde, Cangzhou, Langfang, Hengshui, Taiyuan, Datong, Yangquan, Changzhi, Jincheng, Shuozhou, Jinzhong, Yuncheng, Xinzhou, Linfen, Lüliang, Hohhot, Baotou, Wuhai, Chifeng, Tongliao, Ordos, Hulunbuir, Bayannur, Ulanqab, Shenyang, Dalian, Anshan, Fushun, Benxi, Dandong, Jinzhou, Yingkou, Fuxin, Liaoyang, Panjin, Tieling, Chaoyang, Huludao, Changchun, Jilin, Siping, Liaoyuan, Tonghua, Baishan, Songyuan, Baicheng, Harbin, Qiqihar, Jixi, Hegang, Shuangyashan, Daqing, Yichun, Jiamusi, Qitaihe, Mudanjiang, Heihe, Suihua, Shanghai, Nanjing, Wuxi, Xuzhou, Changzhou, Suzhou, Nantong, Lianyungang, Huai'an, Yancheng, Yangzhou, Zhenjiang, Taizhou, Suqian, Hangzhou, Ningbo, Wenzhou, Jiaxing, Huzhou, Shaoxing, Jinhua, Quzhou, Zhoushan, Taizhou, Lishui, Hefei, Wuhu, Bengbu, Huainan, Ma'anshan, Huaibei, Tongling, Anqing, Huangshan, Chuzhou, Fuyang, Suzhou, Lu'an, Bozhou, Chizhou, Xuancheng, Fuzhou, Xiamen, Putian, Sanming, Quanzhou, Zhangzhou, Nanping, Ningde, Nanchang, Jingdezhen, Pingxiang, Jiujiang, Xinyu, Yingtan, Ganzhou, Ji'an, Yichun, Fuzhou, Shangrao, Jinan, Qingdao, Zibo, Zaozhuang, Dongying, Yantai, Weifang, Jining, Tai'an, Weihai, Rizhao, Linyi, Dezhou, Liaocheng, Binzhou, Heze, Zhengzhou, Kaifeng, Luoyang, Pingdingshan, Anyang, Hebi, Xinxiang, Jiaozuo, Puyang, Xuchang, Luohe, Sanmenxia, Nanyang, Shangqiu, Xinyang, Zhoukou, Zhumadian, Wuhan, Huangshi, Shiyan, Yichang, Xiangyang, Ezhou, Jingmen, Xiaogan, Jinzhou, Huanggang, Xiantao, Tianmen, Qianjiang, Shiyan, Xiangyang, Fushun, Shenyang, Changsha, Zhuzhou, Xiangtan, Hengyang, Shaoyang, Yueyang, Changde, Zhangjiajie, Yiyang, Chenzhou, Yongzhou, Huaihua, Loudi, Guangzhou, Shaoguan, Shenzhen, Zhuhai, Shantou, Foshan, Jiangmen, Zhanjiang, Maoming, Zhaoqing, Huizhou, Meizhou, Shanwei, Heyuan, Yangjiang, Qingyuan, Dongguan, Zhongshan, Chaozhou, Jieyang, Yunfu, Nanning, Liuzhou, Guilin, Wuzhou, Beihai, Fangchenggang, Qinzhou, Guigang, Yulin, Baise, Hezhou, Hechi, Laibin, Haikou, Sanya, Chongqing, Chengdu, Zigong, Panzhihua, Luzhou, Deyang, Mianyang, Guangyuan, Suining, Neijiang, Leshan, Nanchong, Meishan, Yibin, Guang'an, Dazhou, Ya'an, Bazhong, Ziyang, Guiyang, Liupanshui, Zunyi, Anshun, Kunming, Qujing, Yuxi, Baoshan, Zhaotong, Lijiang, Lincang, Xi'an, Tongchuan, Baoji, Xianyang, Weinan, Yan'an, Hanzhong, Ankang, Shangluo, Lanzhou, Jiayuguan, Jinchang, Baiyin, Tianshui, Wuwei, Zhangye, Pingliang, Jiuquan, Qingyang, Dingxi, Longnan, Xining, Yinchuan, Shizuishan, Wuzhong, Guyuan, Zhongwei, Urumqi, Karamay.

8. **The literature should be improved. Authors should incorporate the following papers along with other recent studies on the issue.**

**Illicit financial outflows, informal sector size and domestic resource mobilization in selected African countries**

**External financing for inclusive growth in lower-middle income West African countries: foreign direct investment versus official development assistance**

***Response:***

Thank you very much for your valuable suggestion. The original manuscript lacks recent research on the relevant topic. In response to the two articles you provided, we conducted further studies. And we have integrated the relevant content with the findings of this paper. *External financing for inclusive growth in lower-middle income West African countries: foreign direct investment versus official development assistance.* The literature highlights the significant role of foreign direct investment (FDI) in enhancing inclusivity in middle and low-income countries, suggesting its importance even surpasses that of official development assistance. This aligns with the viewpoint in the original manuscript that the coordinated development of bilateral FDI promotes economic sustainability. *Illicit financial outflows, informal sector size and domestic resource mobilization in selected African countries.* The impact of illicit financial outflows from middle and low-income countries in Africa on domestic resources is a viewpoint lacking in the original manuscript. In response to the suggestions raised regarding this article, we have enriched the conclusions and recommendations in the revised manuscript. The revised part is in “1 Introduction & 6 Conclusions and Recommendations”.

…

The co-ordination advancement of two-way FDI is conducive to the long-term sustainable development of developing countries, and such inclusive growth will strongly promote the optimal allocation of resources and rapid economic growth [1].

…

Lastly, at the macro level, outbound direct investment and foreign direct investment should be controlled to curb illegal capital outflow and provide a better environment for optimizing the allocation of domestic resources [39].

…

9. **The estimation techniques used should be properly discussed with relevant equations stated.**

***Response:***

Thank you very much for your valuable suggestion. In the original manuscript, there was insufficient explanation regarding the selection of the estimation method. In the revised manuscript, detailed justification is provided for employing the two-way fixed effects regression method, along with comprehensive explanations for the relevant equations. The revised part is in “4.1 Benchmark regression”.

…

This paper's data are panel data. Drawing on [36], Before conducting empirical research, we have to use the Hausman test to choose the choice between fixed and random effects. The result shows is chi2 (5) = 46.41, Prob > chi2 = 0.000, we need to use a fixed effect.

…
